# Regarding: ‘Explorative study to identify novel candidate genes related to oxaliplatin efficacy and toxicity using a DNA repair array’

**DOI:** 10.1038/sj.bjc.6605709

**Published:** 2010-06-08

**Authors:** J Pander, H Gelderblom, T van der Straaten, C J A Punt, H-J Guchelaar

**Affiliations:** 1 Department of Clinical Pharmacy and Toxicology, Leiden University Medical Center, PO Box 9600, 2300RC, Leiden, The Netherlands; 2 Department of Clinical Oncology, Leiden University Medical Center, PO Box 9600, 2300RC, Leiden, The Netherlands; 3 Department of Medical Oncology, Radboud University Nijmegen Medical Center, PO Box 9101, 6500 HB, Nijmegen, The Netherlands

**Sir,**

We earlier reported in this journal results from an explorative pharmacogenetic study for the efficacy of second-line treatment of oxaliplatin combined with capecitabine of advanced colorectal cancer (ACC) (Kweekel *et al*, 2009). These results were obtained using a DNA repair array (Asper Biotech, Tartu, Estonia) to identify novel single nucleotide polymorphisms (SNPs) that are associated with progression-free survival (PFS) for oxaliplatin/capecitabine combination therapy (Koopman *et al*, 2007). After correction for multiple testing for five DNA repair pathways investigated, SNPs in the genes encoding ataxia telangiectasia mutated (*ATM* rs1801516) and excision repair cross-complementing group 5 (*ERCC5* rs1047768) were significantly associated with PFS in the final multivariate analysis.

Owing to the explorative nature of the study, we concluded that confirmation was required in a separate cohort of oxaliplatin/capecitabine-treated patients. We, therefore, tested the associations of the same SNPs in the *ATM* and *ERCC5* genes with PFS in patients treated in another cohort – the CAIRO2 study. Blood samples were available of 560 patients who were treated with oxaliplatin combined with capecitabine and bevacizumab, with or without cetuximab, as first-line treatment of ACC (Tol *et al*, 2009). Germline DNA was isolated from peripheral white blood cells by the standard manual salting-out method. We genotyped the *ATM* and *ERCC5* polymorphisms using a Taqman 7500 (Applied Biosystems, Foster City, CA, USA) with pre-designed assays according to the manufacturer's protocol. Negative controls (water) were included. The collection of blood samples for pharmacogenetic research was approved by the local institutional review boards of all participating centres, and all patients gave written informed consent.

The genotype frequencies in the CAIRO2 patients were not significantly different from the earlier study (*P*=0.38 and *P*=0.68 for *ATM* and *ERCC5*, respectively), and were in Hardy–Weinberg equilibrium. However, the frequency of *ATM* homozygote mutants was 1.6% in the CAIRO2 patients *vs* 4.4% in patients in the earlier study.

The results for the associations with PFS are shown in Table 1. As opposed to our initial observation, the *ATM* and *ERCC5* polymorphisms were not significantly associated with PFS in the CAIRO2 patients.

Several reasons could underlie the lack of replication of association. First, our initial results (Kweekel *et al*, 2009) may have been false positive findings. Even though we had corrected for multiple testing, this approach may have been ineffective to correct for false positives. On the other hand, the frequency of *ATM* homozygote mutant patients in the CAIRO2 was lower than in the earlier study, which could have impacted the power to detect the association. However, the HR for PFS was 4.25 (95% CI, 1.45–12.44; homozygote mutants *vs* wild type) in our initial study, whereas it was 0.90 (95% CI, 0.37–2.18) in the CAIRO2 patients, indicating lack of association regardless of genotype frequency.

Second, our initial findings were derived from patients receiving second-line therapy of oxaliplatin combined with capecitabine, whereas CAIRO2 concerns data from first-line therapy with the addition of bevacizumab and cetuximab also. We also recently reported an opposite association of the *FCGR3A* Phe158Val polymorphism with PFS for cetuximab in the first-line setting for ACC compared with results from third-line settings (Pander *et al*, 2010). As the DNA repair array should theoretically be applicable to any platinum-containing regimen, this explanation is less likely for the present finding.

Finally, it is possible that the addition of cetuximab could have negatively influenced the efficacy of oxaliplatin in the cetuximab arm in the CAIRO2 study (Dahan *et al*, 2009; Punt and Tol, 2009), which may have obscured the associations when both treatment arms were combined for analysis. However, the outcome of our analysis did not change when we restricted this to patients treated without cetuximab in the CAIRO2 study (data not shown).

We, therefore, conclude that the *ATM* and *ERCC5* SNPs have no relevant impact on the PFS of oxaliplatin-based therapy for ACC. The negative result of this study underlines the importance of validating and reporting the findings from retrospective explorative studies (Koopman *et al*, 2009).

## Figures and Tables

**Table 1:** Associations of *ATM* (rs1801516) and *ERCC5* (rs1047768) polymorphisms with PFS

	***n***	**Median PFS in months (95% CI)**	**Univariate HR (95% CI)^a^**	***P* a**	**Multivariate HR (95% CI)^a^^,^^b^**	***P* a ^,^ b**
*ATM rs1801516*						
Wild type	371	9.1 (8.3–10.4)	1	—	1	—
Heterozygote	127	12.4 (9.6–13.5)	0.88 (0.70–1.09)	0.245	0.93 (0.75–1.17)	0.543
Homozygote mutant	8	11.8 (7.2-∞)^c^	0.61 (0.27–1.36)	0.225	0.94 (0.42–2.12)	0.881
						
*ERCC5 rs1047768*						
Wild type	180	10.6 (9.1–12.5)	1	—	1	—
Heterozygote	267	9.2 (8.2–10.6)	1.13 (0.93–1.39)	0.227	1.15 (0.93–1.42)	0.194
Homozygote mutant	77	10.1 (8.5–12.2)	0.96 (0.72–1.29)	0.797	0.94 (0.69–1.28)	0.689

Abbreviations: ATM=ataxia telangiectasia mutated; CI=confidence interval; ERCC5=excision repair cross-complementing group 5; HR=hazard ratios; PFS=progression-free survival.

a HR, 95% CI and *P*-values computed using a Cox proportional hazards model with the wild type as reference.

b Covariates included in the multivariate model: age, gender, serum LDH (normal *vs* above normal) and treatment arm (oxaliplatin, capecitabine and bevacizumab *vs* oxaliplatin, capecitabine, bevacizumab and cetuximab).

c The upper limit of the 95% CI for PFS of the ATM homozygote mutants could not be estimated because of the low number of patients.
